# Physician job satisfaction related to actual and preferred job size

**DOI:** 10.1186/s12909-017-0911-6

**Published:** 2017-05-11

**Authors:** Lodewijk J. Schmit Jongbloed, Janke Cohen-Schotanus, Jan C. C. Borleffs, Roy E. Stewart, Johanna Schönrock-Adema

**Affiliations:** 1 Schmit Jongbloed Advies, Hofbrouckerlaan 30, 2341 LP Oegstgeest, The Netherlands; 2 University Medical Center Groningen and University of Groningen, A. Deusinglaan 1, 9713, AV Groningen, The Netherlands

**Keywords:** Job satisfaction, Work ethics, Part time work, Balance work-private

## Abstract

**Background:**

Job satisfaction is essential for physicians’ well-being and patient care. The work ethic of long days and hard work that has been advocated for decades is acknowledged as a threat for physicians’ job satisfaction, well-being, and patient safety.

Our aim was to determine the actual and preferred job size of physicians and to investigate how these and the differences between them influence physicians’ job satisfaction.

**Method:**

Data were retrieved from a larger, longitudinal study among physicians starting medical training at Groningen University in 1982/83/92/93 (*N* = 597). Data from 506 participants (85%) were available for this study. We used regression analysis to investigate the influence of job size on physicians’ job satisfaction (13 aspects) and ANOVA to examine differences in job satisfaction between physicians wishing to retain, reduce or increase job size.

**Results:**

The majority of the respondents (57%) had an actual job size less than 1.0 FTE. More than 80% of all respondents preferred not to work full-time in the future. Respondents’ average *actual* and *preferred* job sizes were .85 FTE and .81 FTE, respectively. On average, respondents who wished to work less (35% of respondents) preferred a job size reduction of 0.18 FTE and those who wished to work more (12%) preferred an increase in job size of 0.16 FTE. Job size influenced satisfaction with *balance work-private hours* most (β = -.351). Physicians who preferred larger job sizes were – compared to the other groups of physicians – least satisfied with *professional accomplishments*.

**Conclusions:**

A considerable group of physicians reported a gap between actual and preferred job size. Realizing physicians’ preferences as to job size will hardly affect total workforce, but may greatly benefit individual physicians as well as their patients and society. Therefore, it seems time for a shift in work ethic.

## Background

Physicians are very dedicated to their work due to a strong believe in the moral benefit and importance of care. This dedication results in long days and hard work. The importance of this work ethic has been passed on for generations and is already emphasized from the beginning of medical training. Consequently, undergraduate students, residents and physicians experience a high workload, which regularly results in reduced job satisfaction or even burnout or resigning from one’s residency or medical profession [1–4].

The burden of a heavy workload is widely acknowledged as a threat for patient safety [5–11]. In order to facilitate physicians to regulate their workload, the European Working Time Directive has been implemented in 1993 [12]. This Directive legally established that physicians do not work more than 48 h a week and no more than 8 h during shifts. Reduction of hours, however, seems difficult to implement in daily practice and physicians’ workload remains high [13, 14]. A possible explanation for this problem is the still existing paradigm that the quality of doctors is dependent on their full-time availability to patients [15]. On a personal level, more and more physicians prefer to reduce their job size to achieve an acceptable workload [16–19]. The question arises, however, whether physicians actually effectuate their preferred job size and if not, how a difference between both relates to job satisfaction.

In general, job satisfaction of physicians is essential for optimal functioning and for quality of care. Higher job satisfaction not only tends to go along with increased well-being and better physical and mental health [20–25], but also with better patient outcomes and higher quality of care [11, 26–32]. A high workload may negatively affect job satisfaction and, hence, the quality of patient care. Therefore, it is important to investigate how job size, as a proxy for workload, is related to job satisfaction.

Our research questions were:

- What are the actual and preferred job sizes of physicians?
- How does actual job size influence physicians’ job satisfaction?
- Are there differences in job satisfaction between physicians who prefer to retain, reduce or increase their job size?

## Methods

### Participants and procedure

The data for this study were retrieved from a larger, longitudinal study at the University of Groningen and University Medical Center Groningen. This longitudinal study encompassed four cohorts of physicians, who started medical training in 1982 (*n* = 166), 1983 (*n* = 167), 1992 (*n* = 171) and 1993 (*n* = 174). Since graduation, 81 out of the 678 physicians (12%) dropped out of the study for several reasons, for instance not practicing medicine anymore, having emigrated, being chronically ill, or because they had deceased. Consequently, 597 graduates were left as potential participants in 2009–2010, of whom 506 (85%) participated. In the current study, the respondents from the cohorts 1982 and 1983 were combined and named as the “older” cohorts (*n* = 265, 52% males and 48% females, average age around 45 years) and those from the cohorts 1992 and 1993 were named as the “younger” cohorts (*n* = 241, 49% males and 51% females, average age around 35 years).

Under the Dutch law, ethical review for this kind of study is not required. All participants were informed about the study and gave their consent to participate. Confidentiality was guaranteed and participation was voluntary.

Over a period of more than a decade, the respondents were interviewed telephonically several times. These interviews focused on various aspects of their work, among which their actual and preferred job size. Besides, at each moment of data collection a specific subject was inquired in-depth. In the last round of data collection (2009/2010), the in-depth subject of inquiry focused on ‘job satisfaction’. The current study focused on data collected in this last round, more specifically on actual and preferred job size and job satisfaction.

### Job size

We asked physicians about their *actual* and their *preferred job size* in Full Time Equivalents (FTE). A full-time job equals 1.0 FTE, which formally corresponds with 48 working hours a week. Part-timers were defined as those working less than 1.0 FTE [16]. Discrepancies between actual and preferred job size were also expressed in FTE. In our analysis, we differentiated between physicians who want to retain, reduce or increase their actual job size in the future.

### Job satisfaction

Job satisfaction is described in sociological literature as a constellation of feelings about various aspects or facets of a job, which are context-specific [33, 34]. To operationalize physicians’ job satisfaction as a multifaceted concept, we explored the medical sociological literature and included those aspects of physician job satisfaction that were most frequently mentioned in literature in our study. This resulted in the inclusion of 13 physician job satisfaction aspects. To warrant that these aspects cover the most important domains of the concept job satisfaction, we related them to the widely acknowledged taxonomy of Ostroff, which has been described as an appropriate conceptual framework for measuring job satisfaction [35–37] and which has been labelled as the most comprehensive classification of work environment perceptions [38] . This framework addresses three key domains of job satisfaction aspects: the *cognitive, affective* and *instrumental* domains. In the cognitive domain, we included (satisfaction with) *opportunities for personal development* [39, 40], *professional accomplishments* [17, 39, 41], *control over work planning, control over work content* [17, 42, 43] and *administrative work* [17, 44]. The affective domain was represented by (satisfaction with) *appreciation from* and *cooperation with colleagues* [17, 19]*, appreciation from* and *cooperation with support personnel* [43–45] and the instrumental domain by (satisfaction with) *cooperation with management* [17, 19], *balance between work and private hours* [43, 44], *appreciation from patients* [17, 43, 44] and *income* [19, 44]. Each aspect of job satisfaction was rated on a 10-point scale (10 = extremely satisfied, 1 = not satisfied at all).

### Data analysis

In this study, *actual* and *preferred* job sizes were included as independent variables and the aspects of job satisfaction as dependent variables. Before deciding which statistical tests to use, we inspected the normality of the score distributions using a normal probability plot of the residuals. The significance level applied was *p ≤* 0.05. Since we found that data displayed approximate normal distributions, it was tenable to use parametric tests. We performed regression analysis to examine the influence of actual job size on the 13 aspects of job satisfaction with cohort and gender added as moderator variables. Unstandardized and standardized regression coefficients were presented with the p values. In addition, we conducted ANOVA to determine any differences in job satisfaction between physicians who wanted to retain, reduce or increase their job size. Statistical significant differences between actual and preferred job size were assessed by a *t*-test for the total group, the gender group and the cohorts group. The means and 95% confidence intervals were calculated to assess the differences. We used the statistical package SPSS for Windows (version 23) [46] and SAS for Windows (version 9.4) [47].

## Results

### Actual and preferred job size

The group difference between physicians’ average *actual* and *preferred* job size was 0.04 FTE (Table 1). Forty-three percent of the respondents had a full-time job, whereas 57% worked part-time (Table 2). Physicians who wanted to work less (35%) preferred an average reduction of 0.18 FTE (CI95% = 0.17–0.19), those who wanted to work more (12%) preferred an average increase of 0.16 FTE (CI95% = 0.14–0.18). Mean difference between full-time and part-time for actual job size was 0.26 FTE (CI95%: 0.24–0.28; *p* < 0.001). Mean difference between full-time and part-time for preferred job size was 0.13 FTE (CI95% : 0.11–0.15; *p* < 0.001).

**Table 1:** Average actual and preferred job size (in Full-Time Equivalents (FTE)) and their difference for male and female physicians of the cohorts 82/83 and 92/93

Gender/Cohort	*N*	Actual job size^a^		Preferred job size		Difference between actual and preferred job size (FTE)
		Mean	95% C.I.^b^	Mean	95% C.I.^b^	
Total	506	0.85	0.84–0.87	0.81	0.80–0.82	0.04
Males	256	0.93	0.92–0.94	0.87	0.85–0.98	0.06
Females	250	0.77	0.75–0.79	0.75	0.73–0.77	0.02
Cohorts 82/83	265	0.84	0.82–0.86	0.80	0.78–0.82	0.04
Males	137	0.94	0.92–0.96	0.87	0.85–0.90	0.07
Females	128	0.74	0.71–0.77	0.72	0.70–0.74	0.02
Cohorts 92/93	241	0.86	0.85–0.88	0.82	0.81–0.84	0.04
Males	119	0.92	0.90–0.94	0.86	0.84–0.87	0.06
Females	122	0.81	0.78–0.84	0.79	0.77–0.81	0.02

^a^1 FTE = full time job ^b^C.I. = confidence interval

**Table 2:** Numbers of full-time (FT) and part-time (PT) working physicians who wish to retain, reduce or increase their actual job size for cohort and gender

		Cohorts 1982/83^a^		Cohorts 1992/93^b^		*N*	*N*
		(*N* = 265)		(*N* = 241)			
		Male	Female	Male	Female		
		(*N* = 137)	(*N* = 128)	(*N* = 119)	(*N* = 122)		
FT who wish to *retain* job size	*N*	51	4	28	8	91 (18%)	217 (43%)
	Act. js^c^	1.0	1.0	1.0	1.0	1.0	
	Pref.js	1.0	1.0	1.0	1.0	1.0	
FT who wish to *reduce* job size	*N*	43	20	38	25	126 (25%)	
	Act. js	1.0	1.0	1.0	1.0	1.0	
	Pref.js	0.81	0.75	0.81	0.83	0.80	
PT who wish to *retain* job size	*N*	22	59	37	59	177 (35%)	289 (57%)
	Act. js	0.78	0.71	0.82	0.71	0.76	
	Pref.js	0.78	0.71	0.82	0.71	0.76	
PT who wish to *reduce* job size	*N*	12	18	10	11	51 (10%)	
	Act. js	0.87	0.82	0.86	0.83	0.83	
	Pref.js	0.74	0.66	0.73	0.69	0.69	
PT who wish to *increase* job size	*N*	9	27	6	19	61 (12%)	
	Act. js	0.73	0.53	0.80	0.66	0.63	
	Pref.js	0.88	0.69	0.94	0.83	0.79	
*N*		137 (27%)	128 (25%)	119 (24%)	122 (24%)	506	

^a^20 years in practice, ^b^10 years in practice, ^c^actual and preferred job size in FTE (mean scores)

With respect to both actual and preferred job size, significant differences were found for gender and cohort. Male physicians worked and preferred to work more than females did (*F* = 152.07, *p* < 0.01 and *F* = 116.96, *p* < 0.01 respectively) (Table 1), and the actual and preferred job sizes of the younger physicians (cohorts 1992 and 1993) were larger than those of physicians from the older cohorts (1982 and 1983) (*F* = 4.51, *p ≤* 0.05 and *F* = 6.60, *p ≤* 0.05 respectively). We found an interaction effect for gender and cohort. Younger females work more and prefer to work more than their older colleagues (*F* = 11.41, *p* < 0.01 and *F* = 18.64, *p* < 0.01).

### The influence of actual job size, cohort and gender on job satisfaction

We found that ‘actual job size’ influenced physicians’ satisfaction with *balance work – private hours* and *control over work planning* negatively (*β* = -0.351, and *β* = -0.151, respectively) (Table 3) and that it influenced their satisfaction with *professional accomplishments* positively (*β* = 0.126). Physicians from the older cohorts were more satisfied with *control over work planning* (*β* = 0.136)*, control over work content* (*β* = 0.094) and *balance work-private hours* (*β* = 0.082) than their colleagues from the younger cohorts. In addition, physicians from the older cohorts were less satisfied with *opportunities for personal development* (*β* = -0.157). Female physicians were less satisfied with *professional accomplishments* (*β* = -0.113), *control over work planning* (*β* = -0.135)*, cooperation with support personnel* (*β* = -0.114) and *balance work-private hours* (*β* = -0.135) than their male colleagues were*.*

**Table 3:** Results of the multiple linear regression analysis to analyse the influence of actual job size, cohort and gender on aspects of job satisfaction

Domain	Dependent variables			Independent variable	Moderator variables	
	Aspects of job satisfaction		Constant	Job size^a^	Cohort^b^	Gender^c^
Cognitive domain	Opportunities for personal development	P	<0.001	0.674	**0.001**	0.949
		B	7.509	0.001	**−0.327**	−0.007
		β		0.021	**−0.157**	−0.003
	Satisfaction with professional accomplishments	p	<0.001	**0.010**	0.840	**0.022**
		B	7.351	**0.006**	−0.015	**−0.189**
		β		**0.126**	−0.009	**−0.113**
	Control over work planning	p	<0.001	**0.002**	**0.002**	**0.006**
		B	7.872	**−0.013**	**0.387**	**−0.386**
		β		**−0.151**	**0.136**	**−0.135**
	Control over work content	p	<0.001	0.321	**0.033**	0.122
		B	7.284	−0.004	**0.225**	−0.184
		β		−0.049	**0.094**	−0.077
	Administrative work	p	<0.001	0.136	0.743	0.632
		B	5.977	−0.007	0.044	0.073
		β		−0.076	0.015	0.024
Affective domain	Appreciation from colleagues	p	<0.001	0.842	0.398	0.152
		B	7.896	−0.001	−0.058	−0.112
		β		−0.010	−0.037	−0.072
	Appreciation from support personnel	p	<0.001	0.437	0.217	0.263
		B	7.494	0.002	0.084	−0.086
		β		0.040	0.056	−0.057
	Cooperation with colleagues	p	<0.001	0.865	0.198	0.285
		B	7.713	0.001	−0.103	−0.097
		β		0.009	−0.057	−0.054
	Cooperation with support personnel	p	<0.001	0.875	0.831	**0.025**
		B	7.508	−0.000	0.015	**−0.173**
		β		−0.008	0.010	**−0.114**
Instrumental domain	Cooperation with management	p	<0.001	0.173	0.585	0.208
		B	7.066	−0.006	−0.073	−0.192
		β		−0.082	−0.029	−0.076
	Balance work-private hours	p	<0.001	**<0.001**	**0.050**	**0.004**
		B	9.388	**−0.028**	**0.211**	**−0.349**
		β		**−0.351**	**0.082**	**−0.135**
	Appreciation from patients	p	<0.001	0.519	0.149	0.654
		B	7.770	−0.001	0.091	−0.032
		β		−0.033	0.065	−0.023
	Income	p	<0.001	0.678	0.145	0.738
		B	7.426	0.002	0.153	0.039
		β		0.021	0.066	0.017

^a^1 FTE = full time job

^b^Cohorts 1982 and 1983 versus cohorts 1992 and 1993 (reference group)

^c^Males (reference group) versus females

*p* = *p* value; B = unstandardized regression coefficient; β = standardized regression coefficient

The bold values represent statistical significance (*p* ≤ 0.05)

### Differences between actual and preferred job size and the impact on job satisfaction

For three aspects of job satisfaction, we found significant differences between physicians who wanted to retain, reduce or increase their job size (Table 4). In comparison with the other groups of physicians, full-time working physicians who wished to reduce their job size were least satisfied with their *balance work-private hours* (*F* = 14.71; *p* < 0.01). Similarly, part-timers who wished to reduce their job size were least satisfied with *cooperation with management* (*F* = 3.18; *p ≤* 0.05), and part-timers who wished to increase their job size were least satisfied with their *professional accomplishments* (*F* = 2.33; *p ≤ 0.05*).

**Table 4:** Average scores on job satisfaction aspects and differences between Full-Time (FT) and Part-Time (PT) working physicians who want to reduce, retain or increase their job size

Job satisfaction aspects	Retain job size^a^		Reduce job size		Increase job size	F^b^	p*
	FT (*N* = 91)	PT (*N =* 177)	FT (*N* = 126)	PT (*N* = 51)	PT (*N* = 61)		
	Mean (SD)^b^	Mean (SD)	Mean (SD)	Mean (SD)	Mean (SD)		
Opportunities for personnel development	7.50 (1.10)	7.40 (0.93)	7.45 (1.21)	7.32 (0.90)	7.56 (1.26)	0.50	0.73
Own professional accomplishments	7.90 (0.77)	7.80 (0.72)	7.90 (0.85)	7.72 (0.70)	7.54 (1.22)	2.33	**0.05**
Control over work planning	6.90 (1.46)	6.93 (1.41)	6.51 (1.45)	6.57 (1.17)	6.70 (1.52)	2.01	0.09
Control over work content	7.19 (1.34)	6.99 (1.13)	6.87 (1.22)	6.90 (1.15)	7.09 (1.25)	1.10	0.36
Administrative work	5.34 (1.60)	5.57 (1.51)	5.27 (1.46)	5.23 (1.41)	5.61 (1.48)	1.20	0.31
Appreciation from colleagues	7.87 (0.69)	7.80 (0.72)	7.73 (0.73)	7.79 (0.66)	7.62 (1.14)	1.01	0.34
Appreciation from support personnel	7.68 (0.89)	7.69 (0.66)	7.64 (0.83)	7.56 (0.61)	7.68 (1.04)	0.32	0.87
Cooperation with colleagues	7.60 (0.95)	7.67 (0.89)	7.68 (0.94)	7.65 (0.65	7.63 (1.04)	0.15	0.96
Cooperation with support personnel	7.47 (0.92)	7.38 (0.67)	7.39 (0.69)	7.24 (0.69)	7.43 (0.96)	0.72	0.58
Cooperation with management	6.53 (1.39)	6.46 (1.03)	6.22 (1.31)	5.68 (1.63)	6.71 (1.27)	3.18	**0.01**
Balance work-private hours	7.05 (1.29)	7.25 (1.16)	6.40 (1.31)	6.58 (1.12)	7.59 (1.13)	14.71	**<0.01**
Appreciation from patients	7.63 (0.76)	7.68 (0.63)	7.69 (0.75)	7.61 (0.61)	7.80 (0.70)	0.69	0.60
Income	7.69 (1.26)	7.70 (0.99)	7.63 (1.35)	7.47 (1.06)	7.56 (1.14)	0.47	0.76

^a^1 FTE = full time job ^b^Degrees of freedom = 4

* Bold values represent statistical significance (*p* ≤ 0.05)

## Discussion

The objective of this study was to gain insight into the actual and preferred job sizes of physicians in relation to job satisfaction. We found that physicians had on average a larger job size than they preferred. Job size relates negatively to job satisfaction, especially to the aspect *balance work-private hours*. Based on the preferred job sizes, a job size of 4 days seems to be ideal for most physicians.

The overall difference between the actual and preferred job sizes of the respondents was small: 0.04 FTE. This outcome may suggest that there is not a real problem. However, some physicians are satisfied with their actual job size (about half), some prefer to work less (about a third) whilst the rest prefers to work more. This means that, on an individual level, the difference between actual and preferred job size may actually be substantial and thus problematic. On average, those who preferred to work less or more wished to reduce and increase their job sizes, on average, with around a day respectively. So, on balance, the difference between actual an preferred job size is rather small which implies that allowing physicians to realize their preferences may not have a major effect on the total workforce. However, attuning actual job sizes to physicians’ preferences may be of vital importance to individual physicians. A better fit between actual and preferred job size may benefit job satisfaction, which – in turn – seems to be in the interest of the health care setting as a whole. High job satisfaction may benefit society through increased well-being and physical and mental health [20–25] and decreased burnout, intention to leave and job or career turnover [32, 48]. Moreover, higher job satisfaction may benefit society through better quality of care, patient outcomes and patient satisfaction [11, 26–32]. Considering the fact that four out of five physicians worked part-time or preferred to work part-time, we conclude that – at least in the Netherlands – the old stereotype of ‘the 24/7 working physician’ [17] does not fit anymore. For the current population of physicians, part-time work seems the solution to reduce the heavy workload and to find a balance between professional and personal life.

Gender differences were found between actual and preferred job sizes with males having larger job sizes. A possible explanation for this outcome may be that from the historical perspective it has been accepted for many years that females have different family commitments than males. In the Netherlands, for example, females predominantly take the responsibility for raising children. Consequently, female physicians may feel a stronger need for working part-time. Our data show indeed that females worked more often part-time than their male colleagues did. However, we also found that around 40% of the full-time working males preferred to reduce their job sizes. A possible reason for this finding may be the changes in (European) society in which males take more and more responsibilities in family commitments. Our data indicate, however, that realizing the preferred job size is more difficult for males than females. It seems very well possible that in a predominantly masculine work context – mainly male specialists and health care employers – managers are less inclined to permit male physicians to work part-time. An often-heard argument against working part-time in the medical field is that it will lead to a higher frequency of handovers, which may hinder continuity of care [5, 49, 50]. We wonder, however, how valid this argument is: We noticed that this argument is seldom used when a physician decides to spend more time on research or teaching which, consequently, also leads to less time for patient care. Since having the preferred job size is beneficial for physicians’ well-being [17, 49] and for quality of care [5, 51], we advise health care managers to appreciate the realization of physicians’ preferred job sizes.

The differences between males and females with respect to actual and preferred job sizes were smaller in the younger cohorts than in the older cohorts. These differences are mainly attributable to the female physicians: female physicians from younger cohorts had relatively larger job sizes and preferred larger job sizes than their female colleagues from the older cohorts did. This outcome is a clear reflection of a societal shift that has taken place in many western countries over the past years: nowadays, it is much more accepted that females have substantial job sizes and that males take up more family commitments.

Our second aim was to examine how actual job size influences physicians’ job satisfaction. Among the job satisfaction aspects that were significantly influenced, the effect of job size on the *balance work-private hours* probably is the most relevant one. The smaller the job size, the more satisfied physicians were with this balance. Our empirical outcomes substantiate earlier notions that a good balance between professional and family life might be an important determinant of job satisfaction [41, 49, 52, 53].

The fact that the mismatch between actual and preferred job size was larger for male than for female physicians may explain why full-time working male physicians who preferred to reduce their job sizes were least satisfied with the *balance between work-private hours*. The need for a better balance between work and their private life seems to be an important reason for preferring a part-time job.

Lower satisfaction with *cooperation with management* seems to be a motive for desiring a further reduction of job size. It may be that the cooperation between physicians and management not only influences physicians’ job satisfaction, but also physicians’ preferences regarding job size. Low satisfaction with *professional accomplishments* seems to be a motive for desiring an increase in job size. Probably, it is difficult to achieve career goals in a too limited job size. We noted that mainly part-time working female physicians wish to increase their job size.

The results of our study suggest that the optimal job size is 4 days a week. Working 4 days a week seems to be sufficient to find a better balance between work and private life, but also to create enough possibilities for professional development. However, further research is needed to explore what the optimal job size is to satisfy these aspects of job satisfaction and which factors influence the optimal balance between job size and these aspects of job satisfaction. We find it important to include gender in these studies, because, roughly speaking, females wish to increase their job sizes, whereas their male colleagues prefer to reduce their job sizes. Other factors that may be relevant for physicians’ preferred or optimal job size are marital status, numbers of children, and other socioeconomic factors.

This study has several strengths. First, we considered job size as a continuous variable. Most studies dichotomized their research population in part-time and full-time working physicians [17, 38, 49, 54]. Because of our approach, we were able to get more detailed information about the exact size of the preferred reduction or increase in job size. Second, we also differentiated between physicians who wished to retain, increase or decrease their job size. This breakdown added to the average outcomes, as it clearly demonstrated substantial mismatches between actual and preferred job sizes of physicians who wanted to change their job sizes.

Third, almost four complete cohorts of graduates were included in our study, which means that the risk of response bias in the outcomes is reduced.

A first limitation of our study is that all graduates studied at the same medical school, which might limit the generalizability of our outcomes. However, our study population is representative of the Dutch population of physicians as the curricula at the Dutch medical schools are very similar and all schools use the Dutch National Blueprint for medical education [55]. Furthermore, more than half of our graduates enrolled in specialty programs of other universities and practice in other parts of the country. The question arises however, how well our outcomes generalize to other countries. It is possible that our results are mainly applicable to the Europe, since the European countries fall under the same legislation. A second limitation is that we restricted our measurement of job satisfaction to the 13 aspects that, in literature, seemed to be most relevant to physicians. It is possible that there are other aspects of job satisfaction that are important to physicians. However, since we were able to address the three domains of Ostroff’s taxonomy – a classification that has been lauded as an all-inclusive, solid integration of the literature [38] – with these 13 aspects we feel confident that we covered the concept job satisfaction of physicians broadly. A third limitation is that job satisfaction also might be influenced by social factors like the marital status, number of children or other socioeconomic factors. However, these confounder variables were not available in the longitudinal dataset from which we retrieved the data for this study. Therefore, further research is necessary to examine the influences of such confounding variables. A fourth limitation is that our data are based on self-assessments. Nevertheless, job satisfaction can only be measured by asking people how satisfied they feel. Finally, our study is restricted to physicians in their begin and middle career stages which might limit the generalizability of our findings to other groups. However, there are indications that our conclusions are also valid for residents and medical students in their practice years: there is evidence that also in these groups a preference for part-time work exists as 15% of male residents and 30% of female residents work part-time [56]. Furthermore, burnout is highly prevalent among residents (27–75%) and medical students (28%–45%) [3, 14], which may also be a sign of too heavy workloads. Future research should investigate whether there also exist differences between the actual and preferred job sizes of medical students and residents and, if so, how these differences influence their job satisfaction.

## Conclusion

Despite the work ethic of long days and hard work that has been advocated for decades, this study shows that the large majority of today’s physicians prefer to work part-time. It seems that it is time for a shift in work ethic, considering the beneficial effects of working part-time as reported in literature on physician health and patient care, and the fact that actualizing all preferred job sizes would hardly affect the total workforce. It may be more ethical towards physicians themselves, their patients, and society as a whole to allow physicians to realize their preferred job size as they know their personal boundaries and needs best. On average, physicians preferred to work 4 days a week. Job size mainly influenced satisfaction with the *balance between work and private hours*. We plea for more acceptance of working part-time, because a better match between actual and preferred job size is important for physicians’ job satisfaction and, thus, on the quality of care.
